# A Microbial World: Could Metagenomic Next-Generation Sequencing Be Involved in Acute Respiratory Failure?

**DOI:** 10.3389/fcimb.2021.738074

**Published:** 2021-10-04

**Authors:** Chunrong Huang, Hong Chen, Yongjie Ding, Xiaolong Ma, Haixing Zhu, Shengxiong Zhang, Wei Du, Hanssa Dwarka Summah, Guochao Shi, Yun Feng

**Affiliations:** ^1^ Department of Respiratory and Critical Care Medicine, Ruijin Hospital, Shanghai Jiao Tong University School of Medicine, Shanghai, China; ^2^ Institute of Respiratory Diseases, Shanghai Jiao Tong University School of Medicine, Shanghai, China; ^3^ Shanghai Key Laboratory of Emergency Prevention, Diagnosis and Treatment of Respiratory Infectious Diseases, Ruijin Hospital, Shanghai, China; ^4^ Department of Respiratory and Critical Care Medicine, The First Hospital of Jiaxing, Jiaxing, China; ^5^ Department of Respiratory and Critical Care Medicine, Poudre D’Or Chest Hospital, Rivière du Rempart, Mauritius

**Keywords:** mNGS, acute respiratory failure, conventional methods, microbial detection, ptNGS

## Abstract

**Background:**

The usefulness of metagenomic next-generation sequencing (mNGS) in identifying pathogens is being investigated. We aimed to compare the power of microbial identification between mNGS and various methods in patients with acute respiratory failure.

**Methods:**

We reviewed 130 patients with respiratory failure, and 184 specimens including blood, bronchoalveolar lavage fluid (BALF), sputum, pleural effusion, ascitic fluid, and urine were tested by mNGS and conventional methods (culture, PCR). We also enrolled 13 patients to evaluate the power of mNGS and pathogen targets NGS (ptNGS) in microbial identifications. Clinical features and microbes detected were analyzed.

**Results:**

mNGS outperformed the conventional method in the positive detection rate of *Mycobacterium tuberculosis* (MTB) (OR, ∞; 95% CI, 1–∞; *P* < 0.05), bacteria (OR, 3.7; 95% CI, 2.4–5.8; *P* < 0.0001), fungi (OR, 4.37; 95% CI, 2.7–7.2; *P* < 0.0001), mycoplasma (OR, 10.5; 95% CI, 31.8–115; *P* = 0.005), and virus (OR, ∞; 95% CI, 180.7–∞; *P* < 0.0001). We showed that 20 patients (28 samples) were detected with *Pneumocystis jirovecii* (*P. jirovecii*) by mNGS, but not by the conventional method, and most of those patients were immunocompromised. Read numbers of *Klebsiella pneumoniae* (*K. pneumoniae*), *Acinetobacter baumannii* (*A. baumannii*), *Pseudomonas aeruginosa* (*P. aeruginosa*), *P. jirovecii*, *cytomegalovirus* (*CMV*), and *Herpes simplex virus 1* (*HSV1*) in BALF were higher than those in other sample types, and the read number of *Candida albicans* (*C. albicans*) in blood was higher than that in BALF. We found that orotracheal intubation and type 2 diabetes mellitus (T2DM) were associated with a higher detection rate of bacteria and virus by mNGS, immunosuppression was associated with a higher detection rate of fungi and virus by mNGS, and inflammatory markers were associated with mNGS-positive detection rate of bacteria. In addition, we observed preliminary results of ptNGS.

**Conclusion:**

mNGS outperformed the conventional method in the detection of MTB, bacteria, fungi, mycoplasma, and virus. Orotracheal intubation, T2DM, immunosuppression, and inflammatory markers were associated with a higher detection rate of bacteria, fungi, and virus by mNGS. In addition, ptNGS results were consistent with the detection of abundant bacteria, fungi, and mycoplasma in our specimens.

## Introduction

Acute respiratory failure (ARF) is a common cause of admission to an intensive care unit (ICU). Given that the management of ARF varies according to the etiology, a clear understanding of underlying pathophysiology is indispensable for managing these patients properly. The causes of ARF can be categorized into lung parenchymal disease, airway obstruction, and neuromuscular dysfunction (Friedman and Nitu, 2018). In the ICU, impaired gas exchange following pulmonary infection has been found to be the most common cause of ARF (Singh Lamba et al., 2016). Patients with ARF tend to experience infections caused by several organisms at the same time (Zarrinfar et al., 2013). Therefore, fast and accurate identification of multiple pathogens remains invaluable to successfully manage these patients.

Metagenomic next-generation sequencing (mNGS) is an advanced and attractive method that allows for the simultaneous detection of bacteria, fungi, viruses, and parasites at multiple sites, including the central nervous system, bloodstream, respiratory, gastrointestinal, prosthetic joint, urinary tract, and ocular (Zhou et al., 2016; Burnham et al., 2018; Ivy et al., 2018; Li et al., 2018b; Simner et al., 2018; Thoendel et al., 2018; Wilson et al., 2018). mNGS holds great promise to be able to transition from being a research tool to being a diagnostic method in clinical microbiology laboratories owing to its quick turnaround time. Previous research also reported that mNGS holds a significantly higher clinical sensitivity and specificity than standard diagnostics. For instance, the ability of mNGS to identify pathogens within 24–48 h is a great advantage over the culture method. Li et al. demonstrated that mNGS showed high sensitivity and specificity for bacterial detection (100.0%, 95% CI: 31.0%–100.0%; 76.5%, 95% CI: 49.8%–92.2%) and fungal detection (57.1%, 95% CI: 20.2%–88.2%; 61.5%, 95% CI: 32.2%–84.9%), when compared with the culture method (Li et al., 2018a). Even though the mNGS method has been used for microbial identification in infectious diseases and severe pneumonia, there have been no studies reporting the applications of mNGS in ARF. Herein, we conducted a single-center study involving hospitalized patients with acute respiratory failure in the ICU to evaluate the clinical performance of the mNGS assay in pathogen detection and compare the power of microbial identi!cation between mNGS and various methods.

## Materials and Methods

### Study Patients and Sample Collection

A total of 130 patients (184 specimens) collected from Ruijin Hospital, Shanghai Jiao Tong University School of Medicine between February 2019 and December 2020 were investigated. Specimens were subjected to regular clinical microbiological assays and mNGS testing in parallel. The study was exempted from ethical review as it was a retrospective study and patient data were anonymized.

The inclusion criteria were in accordance with previous studies, that is a PaO^2^ <60 mmHg in room air. Baseline data including demographic characteristics, comorbidities, immunosuppressive state, treatment process, routine blood, and inflammatory markers were recorded. Different specimens, such as sputum, bronchoalveolar lavage fluid (BALF), blood, pleural effusion, ascitic fluid, and urine, were collected.

### Microbial identifications of Conventional Methods

1) Bacteria, fungi, and mycobacteria were identified using the culture method as follows. Bacteria: specimens were inoculated into media containing blood agar, brain heart infusion broth, chocolate agar, and thioglycollate. The colonies were used to identify bacterial species using the VITEK 2 compact automated system. Fungi: specimens were inoculated into media containing Sabouraud agar and potato glucose agar media. The fungi were identified according to characteristics of the colonies and microscopic characteristics of hyphae and spores. Mycobacteria: sputum was digested with 4% NaOH digestive juice, and fluid specimens were collected after centrifugation for mycobacterium testing. In a 37°C incubator, 0.5 ml of resuspended sputum pellet and 1 ml fluid were inoculated in MGIT. MGIT was put into BACTEC 960 system (BD Microbiology Systems), which could detect automatically. If a positive result appeared, direct acid-fast staining of the smear was done to confirm the positive samples of the mycobacteria. 2) Viruses, *Mycoplasma*, and *Legionella pneumophila* were confirmed using the PCR assay as previously described. Briefly, nucleic acids of *Mycoplasma* and *L. pneumophila* were extracted from each specimen using commercial assays (Qiagen, Germany). They were eluted in distilled water and stored at −20°C for future use. PCR reaction was performed using Applied Biosystems 7500 Real-Time PCR system (Applied Biosystems, USA). Distilled water was used as negative control.

### mNGS Sequencing and Bioinformatic Analysis

For mNGS sequencing, about 300 μl plasma was separated from 3 ml of blood, and DNA was extracted using the TIANamp Micro DNA Kit. With regard to other specimens (BALF, sputum, pleural effusion, ascitic fluid, and urine), 600 μl specimen, enzyme, and 1 g 0.5 mm glass bead were mixed and agitated vigorously at 2,800–3,200 rpm for 30 min, then 300 μl specimens were used for DNA extraction using the TIANamp Micro DNA Kit. DNA was fragmented and constructed by an end-repair, adapter-ligation, and PCR amplification. Quality control of the DNA libraries was performed by Agilent 2100, and then quality qualified libraries were sequenced by MGISEQ-2000 platform (Jeon et al., 2014). Next, raw sequencing data were exposed to quality control, including the removal of low-quality reads (*Q* score cutoff, 20), elimination of human host sequences through mapping to the human reference genome (containing hg19) using Burrows-Wheeler Alignment (Li and Durbin, 2009). The remaining data were aligned to the curated non-redundant Microbial Genome Databases (bacteria, fungi, viruses, parasites). The mapped reads were annotated and the number of species-specific sequences of the specimens were counted.

All of the taxa in the classification reference databases were downloaded from NCBI RefSeq genome database (ftp://ftp.ncbi.nlm.nih.gov/genomes/).

### Pathogen Targets NGS

The multiplex PCR-based targeted gene sequencing technology was performed to identify targeted pathogens, and all of the relevant pathogens explored in sequencing are listed in **Supplementary Table S5**. The workflow was conducted as previously described. Briefly, nucleic acids were extracted according to the instructions of the manufacturer (ZymoBIOMICS™ DNA/RNA Miniprep Kit, Zymo, R2002). Then, a PCR reaction system (including enzyme mix, DNA, RNA, and nuclease-free water) was performed as follows: denaturation at 95°C (3 min); 25 cycles of denaturation at 95°C (20 s), annealing for 20 s, and extension at 60°C for 4.5 min; final extension at 72°C (5 min). The PCR reaction products were purified after amplification. After sequencing adapter assembly, qualified DNA library was sequenced. The reads were filtered, <60 bp reads or non-specific primer binding was removed, and finally, the clean reads were used for sequencing analysis.

### Statistical Analyses

Categorical variables were expressed as percentages, and continuous variables were expressed as mean ± SD if normally distributed. Comparative analysis was conducted by Pearson’s test, Fisher’s exact test, or the McNemar test where appropriate. Data analysis was performed with SPSS 22.0 and Prism. *P <* 0.05 was considered significant.

## Results

### Demographic Characteristics

A total of 130 patients were enrolled in the study. Their demographic characteristics, comorbidities, laboratory examination results, and type of specimen used are summarized in **Supplementary Table S1**. The data showed that 51 out of 130 (39.2%) patients were female, and 79 were male, with a median age of 62.0 years. Among the patients included in the study, 21 (16.15%) patients were immunocompromised, 17 (13.07%) patients had autoimmune diseases, and 29 (22.3%) patients had been diagnosed with cancer. A total of 184 specimens were tested, consisting of 47 sputum, 55 BALF, 74 blood, 6 pleural effusion, 1 ascitic fluid, and 1 urine specimen.

### Pathogens Identified by mNGS

**Figure 1:**
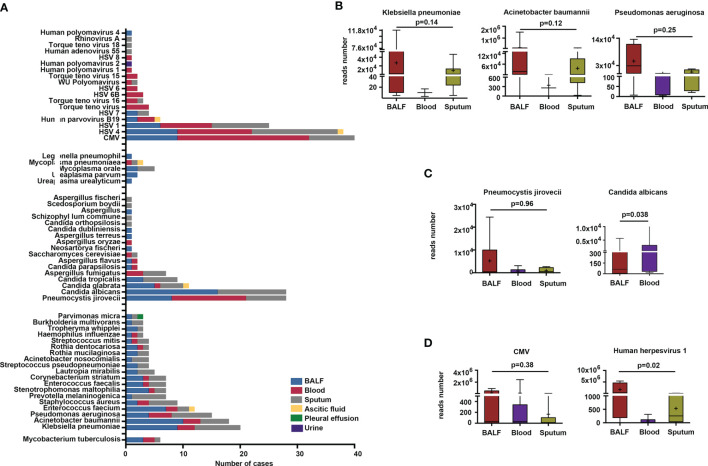
Microbes detected by metagenomic next-generation sequencing (mNGS) method in different sample types. **(A)** Microbes detected in BALF, blood, sputum, pleural effusion, ascitic fluid, and urine samples. **(B–D)** Comparison of reads number of specific bacteria, fungi, and viruses in BALF, blood, and sputum.

**Figure 1** shows the etiological results; a total of 110 microbes were detected by mNGS, including 68 bacteria, *Mycobacterium tuberculosis* (*MTB*), 4 *Mycoplasma*, *Legionella pneumophila*, 17 fungi, and 18 viruses.

We found that the most frequent bacterial pathogens were K. pneumoniae, A. baumannii, Pseudomonas aeruginosa (P. aeruginosa), Enterococcus faecium (E. faecium), Staphylococcus aureus (S. aureus), and Prevotella melaninogenica (P. melaninogenica), all of which were more likely to be detected in BALF and sputum than in other specimen types. Microbes like P. melaninogenica, Lautropia mirabilis (L. mirabilis), Streptococcus pseudopneumoniae (S. pseudopneumoniae), Acinetobacter nosocomialis (A. nosocomialis), Rothia mucilaginosa (R. mucilaginosa), Tropheryma whipplei (T. whipplei), and Burkholderia multivorans (B. multivorans) were present in BALF and sputum only (**Figure 1A**). The results also showed that 10, 19, and 3 bacteria were exclusively detected in BALF, sputum, and blood, respectively (**Supplementary Figure S1**). The most commonly detected fungi were P. jirovecii, C. albicans, C. glabrata, C. tropicalis, and Aspergillus fumigatus (A. fumigatus). P. jirovecii was more commonly detected in BALF, sputum, and blood, while C. albicans and C. tropicalis identifications were limited to BALF and sputum, and A. fumigatus was detected in blood and sputum specimens only (**Figure 1A**). We also found that the most frequently detected viruses were the Epstein–Barr virus (EBV), CMV, and HSV1. Mycoplasma orale (M. orale), Mycoplasma pneumoniae (M. pneumoniae), Ureaplasma parvum (U. parvum), Legionella pneumophilia (L. pneumophilia), and Ureaplasma urealyticus (U. urealyticus) were also detected by mNGS (**Figure 1A**).

Subsequently, we compared the total read numbers of detected microbes in different specimen types. The results show that the sequences of *K. pneumoniae* in BALF (*n* = 9) were higher than those in blood (*n* = 3) and sputum (*n* = 8). The average sequences of *A. baumannii* in BALF (*n* = 10) exceeded the read numbers in blood (*n* = 3) and sputum (*n* = 5). We also noted that the identified sequences of *P. aeruginosa* in BALF outnumbered those in blood and sputum. However, they did not reach statistical difference (**Figure 1B**). Among the fungal sequences, the sequences of *P. jirovecii* in BALF (*n* = 8) were higher than those in blood (*n* = 13) and sputum (*n* = 7). The total reads of *C. albicans* in blood were significantly higher than those in BALF (**Figure 1C**). Besides, we found that, compared with blood and sputum specimens, the viral sequences of *CMV* were more abundant in BALF specimens, even though it was not statistical significant (**Figure 1D**). The read numbers of *HSV1* in BALF were significantly higher than those in blood and sputum (**Figure 1D**).

### Comparison of mNGS and Conventional Methods

**Figure 2:**
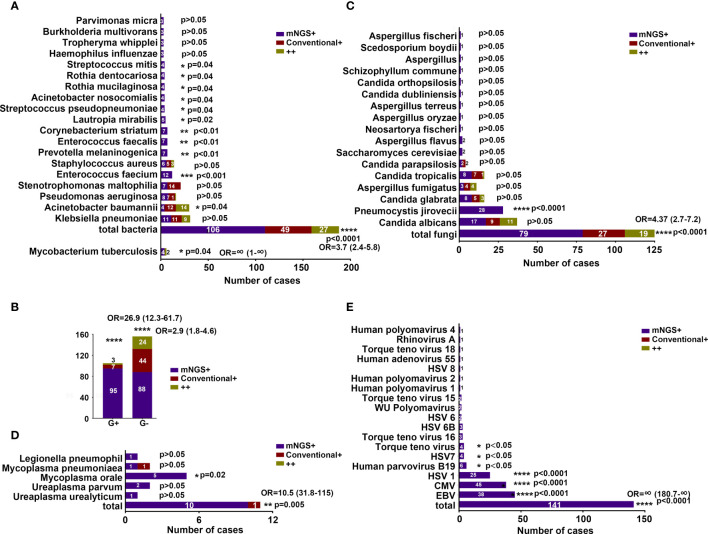
Comparison of positive results by mNGS and conventional methods. **(A)** Comparison of MTB and bacterial positivity by mNGS and conventional methods. **(B)** Comparison of G+ and G− bacterial positivity detected by mNGS and conventional methods. **(C–E)** Comparison of fungal, atypical pathogen, and viral positivity detected by mNGS and conventional methods. *P < 0.05, **P < 0.01, ***P < 0.001, ****P < 0.0001. OR, odds ratio.

Unlike mNGS, among the microbes isolated by conventional methods, *Stenotrophomonas maltophilia* (S. *maltophilia*) was the most common pathogen, followed by *A. baumannii*, *K. pneumoniae*, *P. aeruginosa*, and *S. aureus* (**Figure 2A**). The most frequent fungi detected by the conventional method were *C. albicans*, *C. tropicalis*, *C. glabrata*, *A. fumigatus*, and *C. parapsilosis* (**Figure 2C**). *Mycoplasma pneumoniae* were the only microbes isolated by the conventional method (**Figure 2D**). The viral detection revealed *EBV*, *CMV*, and *HSV1* as the top 3 viruses, as determined by only mNGS (**Figure 2E**).

The comparisons of microbial detection by the mNGS and conventional methods (184 specimens) can be visualized in **Figure 2**. The percentage of mNGS-positive specimens was significantly higher than that of conventional-positive specimens with regard to *MTB* (OR, ∞; 95% CI, 1–∞; *P* < 0.05), bacterial (OR, 3.7; 95% CI, 2.4–5.8; *P* < 0.0001), fungal (OR, 4.37; 95% CI, 2.7–7.2; *P* < 0.0001), mycoplasma (OR, 10.5; 95% CI, 31.8–115; *P* = 0.005), and viral (OR, ∞; 95% CI, 180.7–∞; *P* < 0.0001) detections.

In detail, E. faecium, P. melaninogenica, Enterococcus faecalis (E. faecalis), Corynebacterium striatum (C. striatum), L. mirabilis, S. pseudopneumoniae, A. nosocomialis, R. mucilaginosa, Rothia dentocariosa (R. dentocariosa), and Streptococcus mitis (S. mitis) exhibited a significantly higher detection rate by the mNGS method compared with that noted by the conventional method (P < 0.05) (**Figure 2A**). However, A. baumannii was observed to have a significantly higher yield rate by conventional than by mNGS (P < 0.05) (**Figure 2A**). The frequent K. pneumoniae and P. aeruginosa showed similar detection rate by both methods (**Figure 2A**). We also found that mNGS showed a higher detection rate for both G+ and G− bacteria than the conventional method (**Figure 2B**).

With regard to fungal detection, *Aspergillus flavus* (*A. flavus*) showed a higher detection rate by the mNGS method compared with the conventional method (*P* < 0.05) (**Figure 2C**). Of note, 20 patients (28 specimens) were exclusively detected with *P. jirovecii* by mNGS, and most of the patients were immunocompromised (**Supplementary Table S2**). For patients #6, #56, #60, #69, and #83, blood mNGS identified 23, 240, 164, 72, and 1,097 reads of *P. jirovecii*. The clinical treatment of patients #6, # 69, and #83 was changed, and the treatment of patients #56 and #60 remained unchanged with the continuation of sulfamethazine (SMZ). Sputum mNGS of patients #79 and #90 detected 100 and 31 reads of *P. jirovecii*, the treatment of voriconazole for patient #79 was discontinued, while therapy of patient #90 was not changed. Positive results of BALF mNGS were detected in four patients (#106, 4,627 reads; #114, 790 reads; #115, 1,020 reads; #116, 1,490 reads), of which three patients were added with SMZ therapy. Five patients (#9, #24, #25, #47, #62) had *P. jirovecii* detected in both blood and sputum specimens by mNGS; their treatment was altered, with replacement or discontinuation of broad-spectrum antibiotics and the addition of SMZ. In addition, both BALF and blood mNGS were positive in three patients (#43, #50, #78), and oseltamivir therapy of patient #78 was replaced with SMZ and fluconazole (**Supplementary Table S2**).

The percentage of mNGS-positive specimens was higher than that of the conventional method for *C. albicans* and *C. glabrata*, although the differences were not significant (*P* > 0.05). The percentages of positive specimens were similar in the identification of *A. fumigatus*, *C. tropicalis*, and *C. parapsilosis* by both methods (**Figure 2C**).

Interestingly, some patients were also detected with *M. orale* (*n* = 5 specimens), *CMV* (*n* = 45 specimens), *HSV4* (*n* = 38 specimens), *HSV1* (*n* = 25 specimens), *Human parvovirus B19* (*n* = 6 specimens), *HSV7* (*n* = 4 specimens), and *Torque teno virus* (*n* = 4 specimens); additionally, their yield rate was significantly higher by the mNGS method than by the conventional method (**Figures 2D, E** **)**.

### Implications of mNGS and Conventional Methods in Different Infection Types

**Figure 3:**
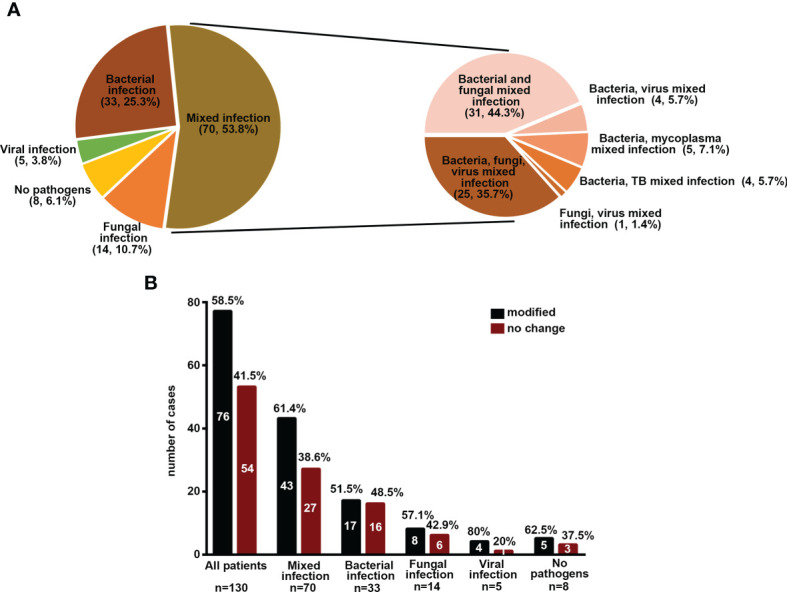
Implications of mNGS and conventional methods in different infection types. **(A)** Percentage of patients with different infection types. **(B)** The percentage of patients with modified treatment regimes.

On the basis of conventional detection results (130 patients), 70 (53.8%), 33 (25.3%), 14 (10.7%), and 5 (3.8%) patients were diagnosed with mixed infection, bacterial infection, fungal infection, and viral infection. With regard to mNGS results, the microbial detection rate (100%) was significantly higher in viral infection, followed by mixed infection (73.9%), fungal infection (63.2%), and bacterial infection (59%) (**Figure 3A**). Among the patients with mixed infection, we noted that 31 (44.3%), 25 (35.7%), 5 (7.1%), 4 (5.7%), 4 (5.7%), and 1 (1.4%) patients had mixed bacterial–fungal, bacterial–fungal–viral, bacterial–viral, bacteria–mycoplasma, bacterial–viral, bacterial–MTB, and fungal–viral infections (**Figure 3A**). The potential detection rate of mNGS in viral infection was the highest, followed by mixed infection, fungal infection, and bacterial infection. However, the positive rate of the conventional method in viral infection was significantly lower than infection from other etiologies (**Supplementary Table S3**).

Among all 130 patients, the pathogens of 58.46% patients were uncovered by an empirical antibiotic regimen, and the antibiotic prescriptions were modified in these patients (**Figure 3B**). However, there was no difference in death rate between patients with modified therapy and those without change (*P* > 0.05) (**Supplementary Figure S2**). Among the patients with different infection types, most patients had modified therapies, especially mixed infection and viral infection. However, the death rate was not significantly different between those with modified therapy and those without change (*P* > 0.05) (**Figure 3B** and **Supplementary Figure S2**, data not shown).

### Inconsistency of Microbial Identifications Between mNGS and the Culture Method

With respect to bacterial identification, mNGS showed positive results in 12 conventional-negative specimens (sputum—62, sputum—75, BALF—82, sputum—100, sputum—104, sputum—108, BALF—121, BALF—125, blood—132, sputum—151, sputum—156, sputum—157). For fungal identification, mNGS yielded positive detection in 10 conventional-negative specimens (blood—11, sputum—75, BALF—82, sputum—88, blood—99, sputum—100, sputum—104, blood—124, sputum—153, BALF—161). In addition, we found in patient #55 (sputum–88) that mNGS and conventional methods produced conflicting data, with mNGS results revealing positive detection of *K. pneumoniae*, while *A. baumannii* and *S. aureus* were detected by the conventional method (**Supplementary Table S4**).

### mNGS Detection Rate Associated With Clinical Characteristics

**Figure 4:**
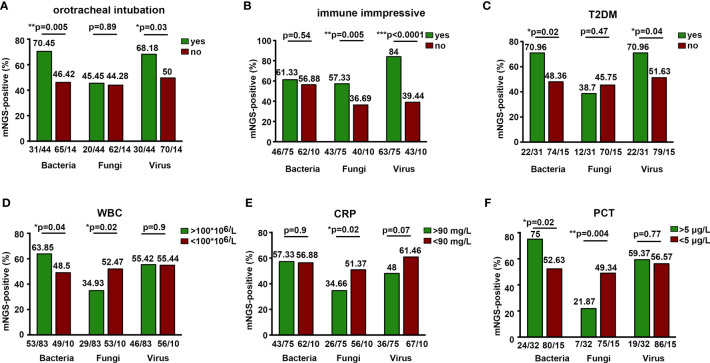
Positive detection rate of mNGS associated with clinical characteristics. *P < 0.05, **P < 0.01, ***P < 0.0001.

mNGS detection rate of bacteria and virus was higher in patients with orotracheal intubation and type 2 diabetes mellitus (T2DM) (**Figures 4A, C** **)**. Immunocompromised patients were found to have a significantly higher detection rate of fungi and virus, indicating the predisposition of fungal and viral infections in patients with immune deficiencies (**Figure 4B**). We also demonstrated a high mNGS-positive rate of bacteria in patients whose detection time was 15 days after being admitted to the hospital (**Supplementary Figure S3B**). However, the clinical outcome (death) and symptom (fever) were not associated with the mNGS microbial detection rate (**Supplementary Figures S3A, C**). We further assessed the effect of laboratory examinations on mNGS detection rate. Blood WBC >100 * 10^6^/L and procalcitonin (PCT) >5 μg/L were related with significantly higher mNGS detection rate of bacteria and lower detection rate of fungi (**Figures 4D, F** **)**. C-reactive protein (CRP) >90 mg/L was associated with lower rate of fungal detection, while there was no relationship between ESR levels and mNGS detection rate (**Figure 4E** and **Supplementary Figure S3D**). These results indicate more common bacterial infections than fungal infections in patients with high WBC, CRP, and PCT. Additionally, further measurement on the influence of IL-1β, IL-2, and IL-6 levels on mNGS detection rate showed that in CNS infections, mNGS detection rate was significantly higher in patients with blood IL-2 >2,000 pg/ml, and IL-6 >40 pg/ml was related with higher mNGS detection rate of fungi or viruses, respectively (**Supplementary Figures S3E–G**).

### Pathogen Targets NGS

A total of 13 patients were enrolled. mNGS and pathogen targets NGS (ptNGS) were performed simultaneously in 13 paired specimens, including #001 blood, #002 sputum, #003 sputum, #004 BALF, #005 sputum, #006 BALF, #007 BALF, #008 sputum, #009 sputum, #010 blood, #011 BALF, #012 sputum, and #013 sputum (**Supplementary Table S6**). We performed the multiplex PCR-based targeted gene sequencing technology to identify targeted pathogens and compared the results of ptNGS and mNGS. All of the relevant pathogens explored in sequencing are listed in **Supplementary Table S5**. ptNGS results were consistent with mNGS in the detection of abundant bacteria, fungi, and mycoplasma in specimens (**Supplementary Table S6**).

For bacterial detection, both ptNGS and mNGS yielded negative results in 3 out of the 13 patients (#001, #010, #013). In patients with positive results, 6 out of the 10 patients were detected with similar abundant species by both methods (#002, #003, #004, #006, #008, #011) (**Supplementary Table S6**).

For fungal detection, ptNGS and mNGS yielded negative results in 7 out of the 13 patients (#001, #004, #005, #008, #009, #011, #012). In patient #007, both methods showed very low sequence. *Pneumocystis jirovecii* and *C. albicans* were detected by ptNGS and mNGS analysis in patient #002. However, *C. parapsilosis* was only found to be positive by mNGS in patient #003 (**Supplementary Table S6**).

For viral detection, ptNGS and mNGS yielded negative results in 3 out of the 13 patients (#004, #007, #009). Both methods only detected very low viral sequences in patients #002 and #006. Both analysis showed low abundance of *CMV* in patient #001 and high abundance of *CMV* in patient #010. However, the two methods showed discrepant results in two patients (#003, #008). In patient #003 (sputum), mNGS presented high sequences of *EBV*, while ptNGS showed low sequences. In patient #008 (sputum), mNGS exhibited low sequences of *Torque teno virus* and *CMV*, while ptNGS demonstrated opposite results (**Supplementary Table S6**).

For mycoplasma detection, mNGS and ptNGS generated negative results in 10 out of 13 patients. Patients #004 (BALF) and #006 (BALF) showed high abundance of *Mycoplasma hominis* by both methods. In patient #007 (BALF), mNGS presented low sequences of *EBV*, while ptNGS showed high sequences (**Supplementary Table S6**).

## Discussion

In this study, we compared the conventional method and mNGS in a pairwise manner, and mNGS proved to be more advantageous when it comes to microbial detection in clinical practice in some aspects. Firstly, mNGS is better than conventional methods in the detection of bacteria, fungi, mycoplasma, and viruses, more specifically *P. jirovecii* and *CMV*. mNGS also demonstrated higher sequencing number of *K. pneumoniae*, *A. baumannii*, *P. aeruginosa*, *P. jirovecii*, *CMV*, and *HSV1* in BALF than in other specimen types. Of note, *P. jirovecii* was exclusively detected by mNGS. Secondly, positive mNGS results assisted in targeted antibiotic prescription, especially when confronted with negative conventional results. Thirdly, ptNGS exhibited a good performance in microbial detection.

mNGS had the advantages of short detection time and unbiased pathogen detection, and these have allowed for its applications in clinical microbial testing (Gu et al., 2019). Steve et al. demonstrated the highly diagnostic accuracy of mNGS through blinded mNGS testing of cerebrospinal fluid (CSF) from 95 patients which revealed 73% sensitivity and 99% specificity compared with the original clinical test results. Subsequent mNGS performance in a validation cohort further showed overall superior sensitivity and specificity to conventional methods (Miller et al., 2019). However, there was no agreement on the advantages of mNGS for etiology diagnosis. For instance, Fang et al. reported mNGS to be a promising method for *non-tuberculous mycobacteria* (*NTM*) identification in BALF due to significantly higher sensitivity (63.64%) than the culture method (36.36%) (Shi et al., 2020). Zhang et al. demonstrated high sensitivity and specificity of mNGS in identifying *S. pneumoniae* in pediatric bacterial meningitis (Zhang et al., 2019). Similarly, previous studies showed the diagnostic value of mNGS in bacterial detection. For instance, mNGS exhibited significantly higher percentage of positive specimens in ventilator-associated pneumonia patients than those detected by conventional testing method (Fang et al., 2020). It also showed apparently higher sensitivity and specificity of pathogen analysis than the culture method in pulmonary infections (Li et al., 2018a). However, with regard to fungal infection, some studies revealed that mNGS showed a satisfying diagnostic performance in infectious diseases and had an overall superior detection rate to culture (OR, 4.0; 95% CI, 1.6–10.3; *P* < 0.01) (Miao et al., 2018), while other research studies noted the comparable detection rate between mNGS and conventional testing methods (OR, 1.42; 95% CI, 0.68–2.97; *P* = 0.46) (Fang et al., 2020). In general, the present study indicated that mNGS outperformed the conventional method in positive detection rate of *MTB*, bacteria, and fungi. In detail, *E. faecium*, *P. melaninogenica*, *E. faecalis*, and *C. striatum* exhibited a higher yield rate by the mNGS method compared with those noted by the conventional method. We also noted the superior feasibility of the conventional method in detecting *A. baumannii*, which is partly contradictory with the research of Fang et al. (2020).

To date, 20 patients in our study were diagnosed with *P. jirovecii* pneumonia (PJP). PJP was diagnosed mostly in AIDS patients, and recent years have witnessed increasing populations in less typical scenarios which were prone to PJP, including hematopoietic stem cell transplant recipients, patients receiving immunosuppressive therapies for autoimmune and inflammatory diseases, and patients with pre-existing lung disease (Khodadadi et al., 2013; Maini et al., 2013; Atwal et al., 2014; Izadi et al., 2014; Avino et al., 2016). Among patients with PJP, most of them were receiving immunosuppressive treatment. *Pneumocystis jirovecii* could be detected in BALF, blood, and sputum by mNGS, but not by the conventional method, and we found that the read numbers in BALF were significantly higher than those in the other two specimen types. Moreover, these results also led to alterations of previous treatment therapies, presenting the great potential of mNGS in guiding treatment options.

Mixed infection is very common in clinical experience and 70% of patients in the current study were diagnosed with mixed infection: 44.3%, 35.7%, 7.1%, 5.7%, 5.7%, and 1.4% patients presented mixed bacterial–fungal, bacterial–fungal–viral, bacterial–viral, bacteria–mycoplasma, bacterial–viral, bacterial–MTB, and fungal–viral infections. mNGS could assess a wide range of organism and microbial profiles, which were often absent in conventional results. Notably, mNGS showed superiority in the simultaneous identification of bacteria, fungi, and viruses when compared with the conventional method, which has also been validated in former research (Fang et al., 2020). Therefore, these results indicated the potential role of mNGS in detecting a broad spectrum of pathogens simultaneously and aiding the clinicians with disease diagnosis and targeted therapeutic schedule. In our study, the therapies of most patients were modified according to the findings of the mNGS. Miao et al. found that 61% patients in their study had initial diagnosis modified based on mNGS findings, and the pathogens were more frequently uncovered (58.6%) by an empirical antibiotic regimen (Miao et al., 2018). In our study, the data showed that more than half of the patients had modified therapies according to the mNGS identification; however, there was no difference of death rate between patients with modified therapy and those without change. Therefore, mNGS could emerge as a promising technology for disease diagnosis on the premise of proper patient selection and data analysis; however, the effect of mNGS-guided therapy on clinical outcomes still needs large-scale studies to delineate.

Apart from mNGS, during recent years, ptNGS was individually developed to facilitate the rapid, cost-effective, sensitive, and specific identification of *MTB*, a major NTM species (Li et al., 2021); specific microorganisms; and antibiotic resistance markers (Hu et al., 2016; Feigelman et al., 2017; Nimmo et al., 2017; Chao et al., 2020). ptNGS uses a selection process before library preparation and sequencing to enrich for microbial sequences of interest. In our study, the enrichment was achieved by multiplex PCR for specific genes, which has been described in blood, sputum, and tissue specimens (Briese et al., 2015; Allicock et al., 2018; Chao et al., 2020; Li et al., 2021). We tried a preliminary exploration on the role of ptNGS in pathogen detection and compared the results of ptNGS and mNGS pairwise in 2 blood specimens, 7 sputum specimens, and 4 BALF specimens (13 patients). We found consistent results of abundant microbial detection by both methods, including *A. baumannii*, *E. faecium*, *P. aeruginosa*, *S. aureus*, *K. pneumoniae*, and *M. hominis*. This indicated the clinical utility of this new approach for pathogen detection, which is also worthy of larger studies to delineate.

Despite these promising results in our study, there were also some limitations. Firstly, the research was limited by the relatively small sample size and the single-center and retrospective nature. For instance, the types of specimens being analyzed in **Figure 2** are mixed and we could not analyze different results from different specimen types. Therefore, a large-scale study is worthy of further research to expect comparisons of positive detections between mNGS and conventional methods in different specimen types. Secondly, the absence of RNA sequencing in our study may lead to reporting bias and false-negative results of RNA viruses. Thirdly, we observed inconsistent results between mNGS and the conventional test. The establishment of the thresholds for mNGS-positive identification is worthy of further research and can assist clinicians to diagnose pathogens. In addition, we noted that the BALF and sputum were from the respiratory tract, which is often mixed with commensal organisms and microbial colonization. For instance, the false-positive results in a previous study suggested the likely colonization of *E. faecium*, *A. baumannii*, and *P. aeruginosa* in the sputum of patients with infectious disease (Miao et al., 2018). The incapability of directly differentiating pathogenic infection and colonization, to some degree, challenged the precision of the mNGS results. Therefore, it not only warned us of the false-positive results of mNGS but also alerted us of the necessity of comprehensive consideration of clinical features and sequencing results in clinical practice.

The clinician should interpret the mNGS results with caution when confronted with the inconsistent results of conventional methods by combining clinical features. mNGS could also emerge as a promising technology for precision diagnosis and tailored therapy in clinical infectious diseases.

## Data Availability Statement

The datasets presented in this study can be found in online repositories. The names of the repository/repositories and accession number(s) can be found below: NCBI and accession PRJNA760800 (https://www.ncbi.nlm.nih.gov/bioproject/PRJNA760800).

## Ethics Statement

Ethical review and approval was not required for the study on human participants in accordance with local legislation and institutional requirements. Written informed consent for participation was not required for this study in accordance with national legislation and institutional requirements.

## Author Contributions

Conception and design of the work: GS and YF. Data analysis: CH, HC, and YD. Data interpretation: HZ, SZ, WD, and XM. Drafting the work or revising it critically for important intellectual content: CH, HS, and YF. All authors contributed to the article and approved the submitted version.

## Funding

This study was supported by Grant 8217010254, 81779925 and 81970020 from the National Natural Science Foundation of China; Grant 2019SY006 from Shanghai Municipal Health Commission; Grant 20dz2261100 from Shanghai Key Laboratory of Emergency Prevention, Diagnosis and Treatment of Respiratory Infectious Diseases; Grant shslczdzk02202 from Shanghai Municipal Key Clinical Specialty; Grant 20dz2210500 from Cultivation Project of Shanghai Major Infectious Disease Research Base; Grant ZH2018QNA48 from Cross Research Funds of Translational Medicine, Grant 2017ZZ02014 from Shanghai Key Discipline for Respiratory Diseases, Grant SHDC12018102 from Shanghai Shenkang Hospital Development Center Clinical Science and Technology Innovation Project.

## Conflict of Interest

The authors declare that the research was conducted in the absence of any commercial or financial relationships that could be construed as a potential conflict of interest.

## Publisher’s Note

All claims expressed in this article are solely those of the authors and do not necessarily represent those of their affiliated organizations, or those of the publisher, the editors and the reviewers. Any product that may be evaluated in this article, or claim that may be made by its manufacturer, is not guaranteed or endorsed by the publisher.
