# Supplementary material for: A Microbial World: Could Metagenomic Next-Generation Sequencing Be Involved in Acute Respiratory Failure?
Source: Front Cell Infect Microbiol. 2021 Oct 4;11:738074. doi: 10.3389/fcimb.2021.738074 (PMC8522648; doi:10.3389/fcimb.2021.738074)
Supplement: Supplementary file 1 [file DataSheet_1.pdf]

Figure S1. Microbes detected by mNGS and conventional methods in different sample types, including BALF, blood, sputum, pleural effusion, ascitic fluid and urine samples.

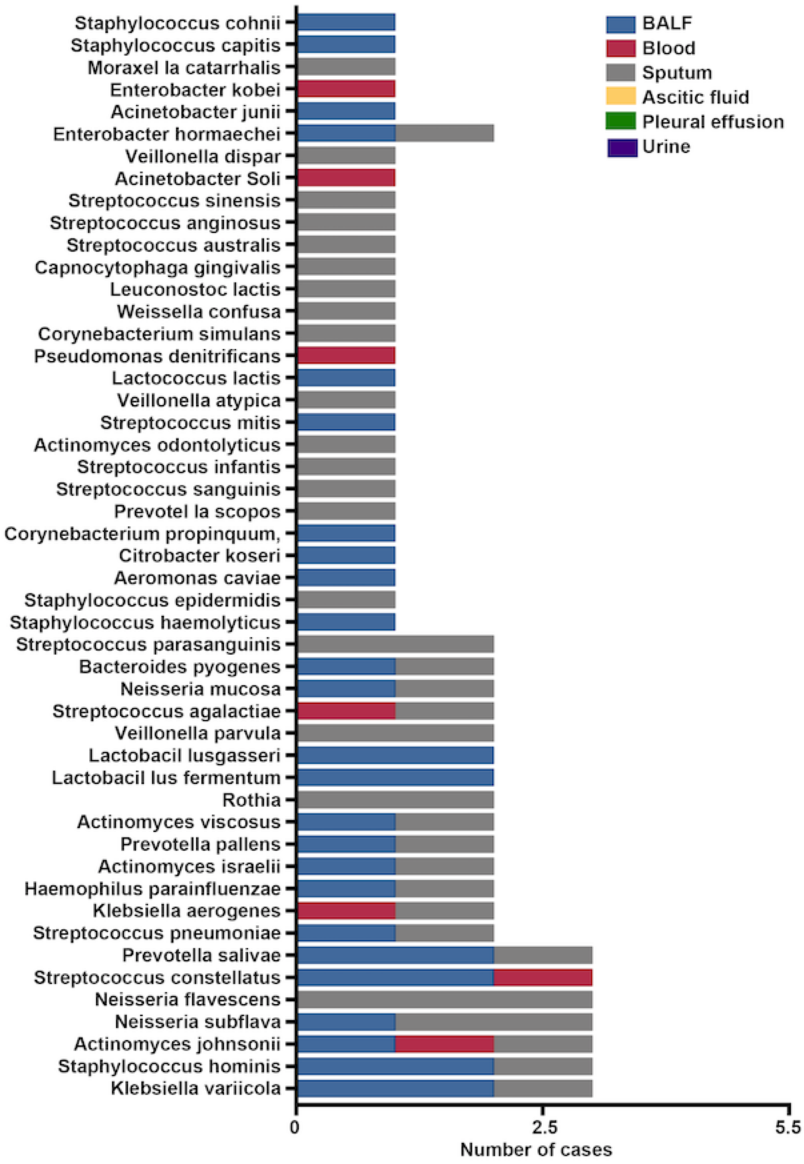


**Figure S2. The difference of death rate between patient with modified therapy and those without changes.**

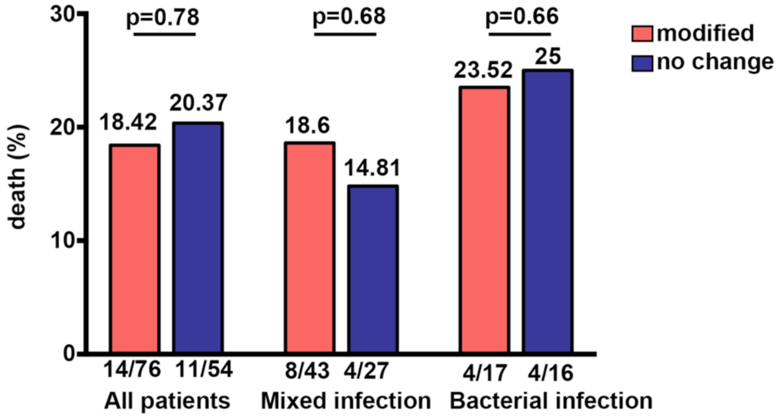


**Figure S3. Positive detection rate of mNGS associated with clinical characteristics.** *$p<0.05$.

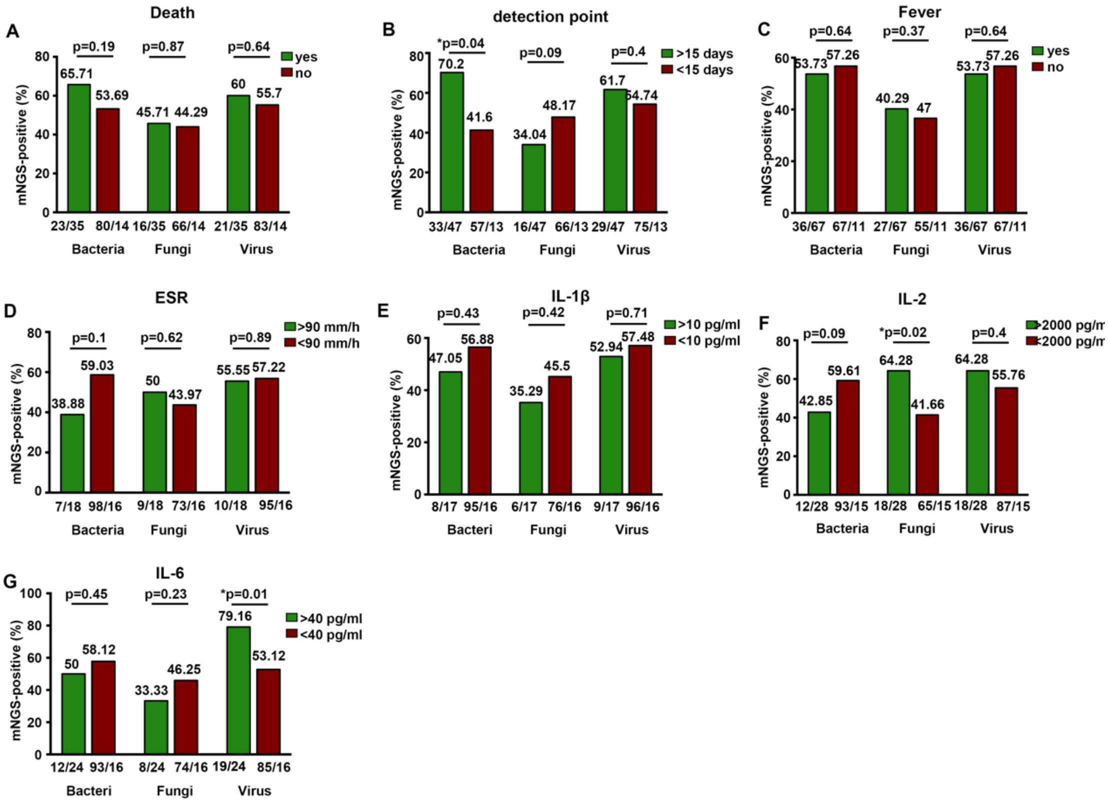

Table S1. Clinical characteristics of 130 patients included.

| Characteristics | Patients |
|---|---|
| Age, years, mean ± SD | 62±16.5 |
| Sex-female, n (%) | 51 (39.2%) |
| **Laboratory parameters** (mean ± SD) | |
| WBC (mean)*, 109/L | 11±6.99 |
| Neutrophil, % | 82.83±14.02 |
| C-reaction protein, mg/L | 86.88±79.94 |
| Erythrocyte sedimentation rate, mm/h | 41.84±32.73 |
| Procalcitonin, ng/ml | 3.8±11.09 |
| **Lymphocytes** (mean ± SD) | |
| CD3, /ul | 615.55±673.42 |
| CD4, /ul | 286.44±220.87 |
| CD8, /ul | 262.85±281.47 |
| CD3+, % | 64.57±16.54 |
| CD3+ CD4+, % | 34.32±12.41 |
| CD3+ CD8+, % | 28.69±15.4 |
| **Cytokines** (mean ± SD) | |
| IL-1β, pg/ml | 8.09±11.62 |
| IL-2, pg/ml | 1741.87±1461.59 |
| IL-6, pg/ml | 57.75±110.06 |
| IL-8, pg/ml | 161.32±375.77 |
| IL-10, pg/ml | 12.79±17.86 |
| TNF-α, pg/ml | 13.97±10.34 |
| **Sample types, n** | **185** |
| Sputum | 47 |
| BALF | 55 |
| Blood | 74 |
| Pleural effusion | 6 |
| Ascitic fluid | 1 |
| Urine | 1 |
| **Comorbidities, n (%)** | |
| Immunosuppression* | 21 (16.15%) |
| Respiratory failure 1 | 99 (75.15%) |
| Respiratory failure 2 | 32 (24.61%) |
| MTB | 4 (3.07%) |
| Autoimmune diseases | 17 (13.07%) |
| Tumor | 29 (22.3%) |

*Immunosuppression was defined as patients with the treatment of ⩾20 mg corticosteroids daily for ⩾14 days; those receiving immunosuppressive therapy.

Table S2. The mNGS results of Pneumocystis jirovecii. for 20 patients without positive conventional result (28 samples).

| Patients ID | BALF reads | Blood reads | Sputum reads | Immune suppression | Changes in treatment |
|---|---|---|---|---|---|
| #6 | NT | 23 | NT | Yes | Added ganciclovir (continuation of SMZ) |
| #9 | NT | 882 | 508 | Yes | Added SMZ and discontinued vancomycin |
| #24 | NT | 1494 | 2280 | NO | Added SMZ, discontinued Voriconazole and vancomycin |
| #25 | NT | 184 | 908 | Yes | Discontinuation of Voriconazole and vancomycin (continuation of SMZ) |
| #43 | 441 | 54 | NT | NO | No change (continuation of SMZ) |
| #47 | NT | 170 | 51 | NO | Added Cefoperazone Sodium and Sulbactam Sodium and Discontinuation of Meropenem (continuation of SMZ) |
| #50 | 12890 | 2 | NT | NO | No change |
| #56 | NT | 240 | NT | NO | No change (continuation of SMZ) |
| #60 | NT | 164 | NT | Yes | No change (continuation of SMZ) |
| #62 | NT | 1328 | 2690 | Yes | Discontinuation of Meropenem (continuation of SMZ) |
| #69 | NT | 72 | NT | Yes | Added Voriconazole (continuation of SMZ) |
| #78 | 11758 | 1361 | NT | Yes | Oseltamivir was replaced with SMZ and Fluconzole |
| #79 | NT | NT | 100 | NO | Discontinuation of Voriconazole |
| #83 | NT | 1097 | NT | Yes | Meropenem and vancomycin changed to Rocephine (continuation of SMZ) |
| #87 | 1408 | NT | NT | NO | Increased dose of SMZ |
| #90 | NT | NT | 31 | NO | No change |
| #106 | 4627 | NT | NT | Yes | Added SMZ |
| #114 | 790 | NT | NT | Yes | Added SMZ |
| #115 | 1020 | NT | NT | Yes | No change (continuation of SMZ) |
| #116 | 1490 | NT | NT | Yes | Ceftazidime fluconazol were replaced with Meropenem and SMZ |

Abbreviations: NT: Not tested. SMZ, Sulfamethazine;

Table S3. The potential pathogen detection rate of mNGS and conventional methods in different types of pulmonary infections.

| Detection rate | No pathogens (n=8 samples) | Bacterial infection (n=39 samples) | Fungal infection (n=19 samples) | Viral infection (n=8 samples) | Mixed infection (n=111 samples) | p value |
|---|---|---|---|---|---|---|
| NGS+ | 0 (0/8) | 59% (23/39) | 63.2% (12/19) | 100% (8/8) | 73.9% (82/111) | 0.0026 |
| Conventional+ | 0 (0/8) | 23.1% (9/39)) | 26.3% (5/19) | 0 (0/8) | 16.2% (29/111) | 0.108 |

Table S4. Inconsistency of microbial identifications between mNGS and conventional method (22 samples).

| Patients ID | Sample ID | mNGS results (bacteria) | mNGS results (fungi) | Conventional results |
|---|---|---|---|---|
| #9 | Blood-11 | | Pneumocystis jirovecii | |
| #9 | Sputum-12 | | Candida albicans, Pneumocystis jirovecii | - |
| #38 | Sputum-62 | Prevotella melaninogenica, Prevotella scopos, Streptococcus sanguinis, Streptococcus infantis, Veillonella atypica, Veil lonella parvula, Rothia mucilaginosa, Rothia aeria | Saccharomyces cerevisiae, Aspergillus fumigatus | Aspergillus fumigatus |
| #41 | Sputum-65 | Neisseria flavescens, Neisseria subflava, Lautropia mirabilis | | - |
| #47 | Sputum-75 | Prevotella pallens, Prevotella melaninogenica, Veillonella parvula, Veillonella atypica, Rothia mucilaginosa, Streptococcus parasanguinis, Streptococcus infantis, CMV | Candida albicans, Pneumocystis jirovecii | - |
| #51 | BALF-82 | Neisseria mucosa, Neisseria subflava, Lautropia mirabilis | Candida albicans | |
| #55 | Sputum-88 | Klebsiella pneumoniae | Candida glabrata, Candida albicans | Acinetobacter baumannii, Staphylococcus aureus |
| #62 | Blood-99 | | Pneumocystis jirovecii | - |
| #62 | Sputum-100 | Staphylococcus epidermidis, Staphylococcus hominis, Neisseria flavescens, Neisseria mucosa, Enterococcus faecium, Enterococcus durans, Veillonella parvula, Veillonella dispar, | Pneumocystis jirovecii | - |

| | | | | |
|---|---|---|---|---|
| | | Treponema denticola, Treponema socranskii, Ochrobactrum intermedium, Streptococcus anginosus | | |
| #65 | Sputum-104 | Prevotella histicola, Prevotella melaninogenica, Veillonella parvula, Veillonella dispar Streptococcus parasanguinis, Streptococcus australis, Actinomyces graevenitzii, Actinomyces radicidentis, Atopobium parvulum, Corynebacterium striatum, Corynebacterium kroppenstedtii, Campylobacter concisus, Scardovia wiggsiae, Enterococcus faecium, Rothia mucilaginosa | Candida albicans | - |
| #66 | Blood-105 | Prevotella histicola, Prevotella melaninogenica, Veillonella parvula, Veillonella dispar, Streptococcus parasanguinis, Streptococcus australis, Actinomyces graevenitzii, Actinomyces radicidentis, Atopobium parvulum, Corynebacterium striatum, Corynebacterium kroppenstedtii, Campylobacter concisus, Scardovia wiggsiae, Enterococcus faecium, Rothia mucilaginosa | | Staphylococcus epidermidis |
| #67 | Sputum-108 | Enterococcus faecalis | | - |
| #73 | Blood-116 | - | - | Staphylococcus epidermidis |
| #76 | BALF-121 | Klebsiella pneumoniae | - | - |
| #78 | Blood-124 | | Pneumocystis jirovecii | - |

| | | | | |
|---|---|---|---|---|
| #78 | BALF-125 | Tropheryma whipplei | - | - |
| #83 | Blood-132 | Tropheryma whipplei | - | - |
| #99 | Sputum-151 | Moraxella catarrhalis | - | - |
| #100 | Sputum-153 | - | Candida albicans | - |
| #102 | Sputum-156 | Enterococcus faecium, Enterococcus faecalis | - | - |
| #103 | Sputum-157 | Prevotella melaninogenica, Veillonella atypica, Haemophilus sputorum, Fusobacterium periodonticum, Fusobacterium nucleatum, Porphyromonas somerae | - | - |
| #106 | BALF-161 | | Pneumocystis jirovecii | |

Table S5. All of the target pathogens covered in ptNGS and mNGS sequencing

| Gram-positive bacteria | Gram-negative bacteria | Fungi | Virus | Parasite | Other pathogens |
|---|---|---|---|---|---|
| Actinomyces | Acinetobacter | Pneumocystis | Adenovirus | Pork tapeworm | Chlamydia pneumoniae |
| Israeli | baumannii | jirovecii Aspergillus | human bocavirus | Beef tapeworm | Chlamydia psittaci |
| Enterococcus | Klebsiella pneumonia | flavus | CMV | Fasciolopsis buski | Mycoplasma |
| faecalis | Haemophilus | Aspergillus fumigatu | EBV | American hookworm | pneumoniae |
| Enterococcus | influenzae | Aspergillus niger | epidemic encephalitis B virus | Filaria bancrofti | |
| Faecium | Pseudomonas | Aspergillus terreus | pseudolymphocytic | Schistosomiasis | |
| Mycobacterium | aeruginosa | Candida albicans | choriomeningitis virus | japonica | |
| intracellulare | Stenotrophomonas | Candida glabrata | mumps virus | Toxoplasma gondii | |
| Mycobacterium | maltophilia | Candida tropicalis | Coxsackievirus A16 | Toxoplasma | |
| tuberculosis | Bordetella pertussis | Cuyitococcus | Coxsackievirus A6 | | |
| complex | Burkholderia cepacia | Neofonmans | Coxsackievirus B | | |
| Nocardia | Citrobacter freundii | Histoplasmosis | Enterovirus 71 | | |
| asteroids | Enterobacter cloacae | capsulati | Parainfluenze virus 1 | | |
| Nocardia | Escherichia coli | Candida krusei | Parainfluenze virus 3 | | |
| brasiliensis | Enterobacter | Penicillium Marneffei | Parainfluenze virus 4 | | |
| Nocardia caviae | aerogenes | Mucorcircinelloides | Human Coronavirus 229E | | |
| Staphylococcus | Klebsiella oxytoca | Mucor pusillus | Coronavirus HKU1 | | |
| aureus | Legionella | Mucor Asarum | Coronavirus NL63 | | |
| Streptococcus | pneumophila | Candida parapsilosis | Coronavirus OC43 | | |
| constellatus | Moraxella catarrhalis | | Human metapneumovirus | | |
| Pneumococcal | Neisseria meningitidis | | Respiratory Syncytial Virus A | | |
| pneumonia | Serratia marcescans | | Respiratory Syncytial Virus B | | |

| | | |
|---|---|---|
| Streptococcus pyogenes<br>corynebacterium striatum | Bartonella henselae | Human rhinovirus<br>Influenza A virus<br>Influenza B virus<br>Measle virus<br>Rubella virus<br>COVID-19<br>Rabies virus |

Table S6. Comparisons of ptNGS and mNGS results in 13 patients (13 paired samples).

| Patients ID | Sample | Bacteria | Fungi | Virus | Mycoplasma |
|---|---|---|---|---|---|
| #001 Blood | mNGS (reads) | - | - | CMV (94)<br>Torque teno virus (13)<br>Torque teno virus 19 (8) | - |
| | ptNGS (copies/ml) | - | - | CMV (<1x$10^2$) | - |
| #002 Sputum | mNGS (reads) | **Acinetobacter baumannii (8444)** | **Pneumocystis jirovecii (731)**<br>**Candida albicans (464)**<br>Candida glabrata (32)<br>Aspergillus fumigatus (1) | EBV (5) | - |
| | ptNGS (copies/ml) | **Acinetobacter baumannii (6x$10^6$)** | **Candida albicans (2x$10^5$)**<br>**Pneumocystis jirovecii (2x$10^3$)** | CMV (2)<br>HSV (1) | - |
| #003 Sputum | mNGS (reads) | **Enterococcus faecium (146211)** | Candida parapsilosis (79053) | EBV (25407) | - |
| | ptNGS (copies/ml) | **Enterococcus faecium (5x$10^6$)**<br>Enterococcus faecalis (2x$10^6$)<br>pseudomonas alcaligenes (5) | Candida lusitaniae (4) | EBV (79) | |
| #004 BALF | mNGS (reads) | **Pseudomonas aeruginosa (4470)**<br>**Staphylococcus aureus (3861)**<br>**Klebsiella pneumoniae (2438)**<br>**Acinetobacter baumannii (490)**<br>Enterococcus faecalis (599)<br>Pseudomonas denitrificans (485)<br>Klebsiella quasipneumoniae (138)<br>Staphylococcus argenteus (97) | - | - | Mycoplasma hominis (1816) |

| | | | | | |
|---|---|---|---|---|---|
| | ptNGS (copies/ml) | **Staphylococcus aureus (8x10$^5$)**<br>**Pseudomonas aeruginosa (5x10$^5$)**<br>**Klebsiella pneumoniae (3x10$^5$)**<br>**Acinetobacter baumannii (8x10$^3$)**<br>Enterococcus faecalis (3x10$^3$)<br>Corynebacterium striatum (1x10$^6$)<br>Enterococcus faecium (1) | - | - | Mycoplasma hominis (8x10$^5$) |
| #005 Sputum | mNGS (reads) | **Klebsiella pneumoniae (192525)**<br>Klebsiella variicola (7087)<br>Pyramidobacter piscolens (1062)<br>Enterobacter roggenkampii (640)<br>Enterobacter hormaechei (42)<br>Escherichia coli (90) | - | HSV (8) | |
| | ptNGS (copies/ml) | **Klebsiella pneumoniae (1x10$^5$)**<br>Haemophilus parainfluenzae (1x10$^3$)<br>Enterococcus faecalis (<1x10$^2$) | - | HSV7 (1x10$^3$)<br>CMV (43)<br>HSV 6B (16) | |
| #006 BALF | mNGS (reads) | **Acinetobacter baumannii (80519)**<br>**Pseudomonas aeruginosa (1139)**<br>**Staphylococcus aureus (281)**<br>Dermabacter hominis (203)<br>Proteus mirabilis (15) | Candida tropicalis (7) | - | Mycoplasma hominis (7824) |
| | ptNGS (copies/ml) | **Acinetobacter baumannii (1x10$^6$)**<br>**Pseudomonas aeruginosa (1x10$^5$)**<br>**Staphylococcus aureus (6x10$^4$)**<br>Streptococcus pneumoniae (10) | - | Rhinovirus A (2) | Mycoplasma hominis (1x10$^6$) |

| | | | | | |
|---|---|---|---|---|---|
| #007<br>BALF | mNGS (reads) | **Klebsiella pneumoniae (249)**<br>Pseudomonas aeruginosa (122)<br>Achromobacter xylosoxidans (104)<br>Proteus mirabilis (33) | Candida tropicalis (2)<br>Candida albicans (1) | - | Mycoplasma hominis (2) |
| | ptNGS (copies/ml) | **Staphylococcus aureus (4x$10^6$)**<br>Haemophilus parainfluenzae (2x$10^6$)<br>Pseudomonas aeruginosa (2x$10^6$)<br>Klebsiella pneumoniae (1x$10^6$)<br>Stenotrophomonas maltophilia (85)<br>enterobacteria (3) | Candida tropicalis (3) | - | Mycoplasma hominis (5x$10^6$) |
| #008<br>Sputum | mNGS (reads) | **Pseudomonas aeruginosa (14018)**<br>Streptococcus constellatus (27) | - | Torque teno virus (13)<br>CMV (3)<br>Torque teno mini virus 18 (3) | - |
| | ptNGS (copies/ml) | **Pseudomonas aeruginosa (4x$10^6$)**<br>Stenotrophomonas maltophilia (4x$10^5$)<br>Acinetobacter baumannii (4) | - | Torque teno virus (6x$10^6$)<br>CMV (3x$10^4$) | - |
| #009<br>Sputum | mNGS (reads) | **Klebsiella pneumoniae (4012)**<br>Burkholderia multivorans (723)<br>Klebsiella variicola (96) | - | - | - |
| | ptNGS (copies/ml) | **Corynebacterium striatum (2x$10^5$)**<br>Stenotrophomonas maltophilia (1x$10^5$)<br>Klebsiella pneumoniae (3x$10^4$)<br>Pneumococcal pneumonia (3x$10^4$) | - | - | - |

| | | | | | |
|---|---|---|---|---|---|
| | | Actinomyces Israeli (1x10$^{2}$) | | | |
| #010 Blood | mNGS (reads) | - | **Pneumocystis jirovecii (32)** | CMV (2499) | - |
| | ptNGS (copies/ml) | - | **Pneumocystis jirovecii (1x10$^{2}$)** | CMV (5x10$^{4}$)<br>EBV (33) | - |
| #011 BALF | mNGS (reads) | **Staphylococcus aureus (1759)**<br>**Pseudomonas aeruginosa (979)** | - | - | - |
| | ptNGS (copies/ml) | **Pseudomonas aeruginosa (7x10$^{5}$)**<br>**Staphylococcus aureus (8x10$^{4}$)**<br>Acinetobacter baumannii (1x10$^{6}$)<br>Enterococcus faecalis (1x10$^{3}$)<br>Pseudomonas alcaligenes (2) | - | CMV (1x10$^{4}$)<br>HSV (71)<br>EBV (3) | - |
| #012 Sputum | mNGS (reads) | Enterococcus faecium (4) | - | - | - |
| | ptNGS (copies/ml) | - | - | CMV (3x10$^{5}$)<br>HSV7 (1x10$^{4}$) | - |
| #013 Sputum | mNGS (reads) | - | **Candida albicans (1421)** | Human herpesvirus 1 (192)<br>EBV (23) | - |
| | ptNGS (copies/ml) | - | **Candida albicans (1x10$^{3}$)**<br>Pneumocystis jirovecii (1x10$^{3}$) | EBV (4x10$^{5}$)<br>Human herpesvirus 7 (7x10$^{4}$)<br>Human herpesvirus 1 (2x10$^{4}$)<br>Human herpesvirus 6B (8x10$^{3}$)<br>CMV (9x10$^{2}$)<br>HSV 6A (2) | - |
